# Hybrid ablation of atrial fibrillation: A unilateral left‐sided thoracoscopic approach

**DOI:** 10.1111/jocs.17144

**Published:** 2022-11-09

**Authors:** Claudia A. J. van der Heijden, Vanessa Weberndörfer, Justin G. L. M. Luermans, Sevasti‐Maria Chaldoupi, Sander M. J. van Kuijk, Mindy Vroomen, Elham Bidar, Jos G. Maessen, Laurent Pison, Mark La Meir, Bart Maesen

**Affiliations:** ^1^ Department of Cardiothoracic Surgery Maastricht University Medical Centre Maastricht The Netherlands; ^2^ Cardiovascular Research Institute Maastricht Maastricht The Netherlands; ^3^ Department of Cardiology Maastricht University Medical Centre Maastricht The Netherlands; ^4^ Department of Clinical Epidemiology and Medical Technology Assessment Maastricht University Medical Center Maastricht The Netherlands; ^5^ Department of Cardiology Hospital East Limburg Genk Belgium; ^6^ Department of Cardiac Surgery UZ Brussel Brussels Belgium

**Keywords:** atrial fibrillation, hybrid ablation, unilateral left‐sided approach

## Abstract

**Background:**

Hybrid ablation (HA) of atrial fibrillation (AF) combines minimally invasive thoracoscopic epicardial ablation with transvenous endocardial electrophysiologic validation and touch‐up of incomplete epicardial lesions if needed. While studies have reported on a bilateral thoracoscopic HA approach, data on a unilateral left‐sided approach are scarce.

**Aim:**

To evaluate the efficacy and safety of a unilateral left‐sided thoracoscopic approach.

**Methods:**

Retrospective analysis of a prospectively gathered cohort of all consecutive patients undergoing a unilateral left‐sided HA for AF between 2015 and 2018 in the Maastricht University Medical Centre.

**Results:**

One‐hundred nineteen patients were analyzed (mean age 64 ± 8, 28% female, mean body mass index 28 ± 4 kg/m^2^, median CHA_2_DS_2_‐VASc Score 2 [1–3], [longstanding]‐persistent AF 71%, previous catheter ablation 44%). In all patients, a unilateral left‐sided HA consisting of pulmonary vein (PV) isolation, posterior left atrial (LA) wall isolation, and LA appendage exclusion was attempted. Epicardial (*n* = 59) and/or endocardial validation (*n* = 81) was performed and endocardial touch‐up was performed in 33 patients. Major peri‐operative complications occurred in 5% of all patients. After 12 and 24 months, the probability of being free from supraventricular tachyarrhythmia recurrence was 80% [73–87] and 67% [58–76], respectively, when allowing antiarrhythmic drugs.

**Conclusion:**

Unilateral left‐sided hybrid AF ablation is an efficacious and safe approach to treat patients with paroxysmal and (longstanding) persistent AF. Future studies should compare a unilateral with a bilateral approach to determine whether a left‐sided approach is as efficacious as a bilateral approach and allows for less complications.

**Abbreviations**
AAD: antiarrhythmic drug
AF: atrial fibrillation
AT: atrial tachycardia
HA: hybrid ablation
LA: left atrium
LAA: LA appendage
pAF: paroxysmal AF
persAF: persistent AF
PV: pulmonary vein
PVI: PV isolation
SR: sinus rhythm

## 1 INTRODUCTION

Thoracoscopic ablation for atrial fibrillation (AF), including hybrid AF ablation, is recognized as a valid treatment option for patients with symptomatic, drug‐refractory paroxysmal, or persistent AF.^1^ The standard hybrid ablation (HA) procedure combines minimally invasive bilateral thoracoscopic epicardial ablation with transvenous endocardial electrophysiologic validation and touch‐up of incomplete epicardial lesions if needed.^2^ The results of HA are promising when performed by an experienced team, with high success percentages even after 3 years of follow‐up (freedom from atrial arrhythmia recurrence of 80% for paroxysmal AF and 79% for more persistent forms).^3^ Furthermore, when comparing HA with catheter ablation (CA) in a meta‐analysis, the outcome following HA is superior to CA for persistent and long‐standing persistent AF until 12 months (70.7% vs. 49.9%, *p* < .001).^4^

More recently, we have changed our surgical technique to a unilateral left‐sided thoracoscopic approach.^5^ The possible advantage of a unilateral approach is the reduced risk of complications on the contralateral side, while still performing the same lesion set as in a bilateral approach. Although studies have reported on the bilateral HA approach,^2^ cohort data on a unilateral left‐sided approach are scarce. Therefore, we aimed to evaluate the efficacy and safety of a unilateral left‐sided thoracoscopic approach in all consecutive patients referred for HA in our center.

## 2 PATIENTS AND METHODS

### 2.1 Study population and design

In this retrospective analysis of a prospectively gathered cohort, we analyzed all patients who underwent a unilateral left‐sided thoracoscopic HA between January 2015 and December 2018 at the Maastricht University Medical Centre. The study was approved by the Institutional Review Board (IRB) and Ethics Committee (METC 2019‐1430) in accordance with IRB guidelines and patient informed consent was waived. The study complies with the ethical principles of the Helsinki Declaration.

### 2.2 Hybrid Ablationprocedure

All HA procedures were performed by an experienced team consisting of two surgeons (B. M. and M. L. M) and two electrophysiologists (J. L. and L. P.). The technique of unilateral left‐sided HA has been described previously.^5^ In brief, left‐sided thoracoscopic ablation consisting of left and right pulmonary vein (PV) isolation using a biparietal bipolar radiofrequency clamp (Isolator, AtriCure) was performed, followed by ablation of the roof and inferior lines (Coolrail, AtriCure) to create the box lesion. In all patients, closure of the left atrial appendage (LAA) was attempted. Finally, epicardial and/or endocardial electrophysiological validation of the entrance and exit block of the PVs and the box was attempted and if necessary, additional touch‐up of unintended gaps was performed. For patients with a left atrial (LA) flutter or ‐tachycardia, additional ablation thereof was performed endocardially, if inducible. A left isthmus line was made in patients developing a mitral isthmus‐dependent atrial flutter during the procedure and a cavotricuspid isthmus (CTI) line if the patient presented with right atrial (RA) dilatation or a typical atrial flutter.

### 2.3 Outcomes and follow‐up

The primary outcome was defined as freedom from any supraventricular tachyarrhythmia ≥30 s until 24 months of follow‐up, allowing antiarrhythmic drugs (AADs), after a 3 months blanking period.^1^ Discontinuation of AADs after the blanking period was encouraged if the patient appeared to be arrhythmia free, though the final decision was left to the discretion of the referring cardiologist. Secondary outcomes included peri‐ and postoperative complications until 24 months: bleeding, cardiac tamponade, conversion to sternotomy, hemothoraxor pleural effusion requiring drainage, myocardial infarction,^6^ mortality, pacemaker implantation, pericarditis (either stable hemodynamics requiring optimal medication therapy or unstable hemodynamics requiring pericardiocentesis), phrenic nerve injury, pneumothorax (after removal of chest tubes), and stroke.

All patients were encouraged to visit the outpatient clinic at 3, 12, and 24 months. Rhythm follow‐up consisted of 24, 48‐h, or 7‐days Holter monitoring (dependent on the preference of the physician), read‐out of implanted devices, or by 12‐lead ECG monitoring, conform our standard of care. In case of AF recurrence, patient‐tailored treatment followed conform routine care.

### 2.4 Statistical analysis

Continuous variables were expressed as mean ± standard deviation or median and interquartile range, while categorical variables as count and relative frequencies. A Pearson's *χ* ^2^ test or Fisher's exact test was performed to compare categorical variables between both groups, whereas a Student's *t*‐test or Mann–Whitney *U* test (dependent on data distribution) was used for the comparison of continuous variables. One‐way analysis of variance was used for the comparison of continuous variables between multiple groups. Next, a Kaplan–Meier curve was plotted to estimate the probability of being free from any supraventricular recurrence within the course of 24 months. The log‐rank test was used to test for differences in efficacy outcome throughout the whole follow‐up period between patients undergoing endocardial and epicardial validation only for the whole group, paroxysmal AF only and persistent AF only. A *p* value <.05 was considered statistically significant and all analyses were performed using SPSS version 25.0 (SPSS Inc.) and R version 4.1.2.

## 3 RESULTS

### 3.1 Study population

In total, 119 patients were analyzed. Patients were on average 64 ± 8 years old, 28% was female, and the mean body mass index (BMI) was 28 ± 4 kg/m^2^ with a median CHA_2_DS_2_‐VASc Score of 2 ^1^, ^2^, ^3^ (Table 1). Four percent had a history of a hemorrhage, 13% had obstructive sleep apnea and 8% had vascular disease. More patients had (longstanding)‐persistent AF (71%) than paroxysmal AF (29%) and the median history of AF duration was 61 [25–125] months. Forty‐five patients (38%) were also known with an AFL and/or AT and 53 patients (44%) underwent previous CA for AF or AFL. The mean LVEF and LA volume index were 54 ± 9% and 50 ± 16 ml/m^2^, respectively. The mean RA volume index was 38 ± 14 ml/m^2^. When comparing baseline characteristics of the patients who underwent epicardial validation only (*n* = 33) with those who underwent (at least) endocardial validation (*n* = 81), patients were older (66 ± 7 vs. 62 ± 8, *p* = .014), mean BMI was lower (26 ± 3 vs. 28 ± 4 kg/m^2^, *p* = .019) and more patients had a previous hemorrhage (12% vs. 0%, *p* = .002). All other characteristics were comparable between both groups (Table 1).

**Table 1:** Baseline characteristics of the total population compared with patients who received epicardial validation only versus at least endocardial validation.

Patient characteristics	All patients (*n* = 119)	Epicardial validation only (*n* = 33)	Endocardial validation (*n* = 81)	*p* Value
Age (years)	64 ± 8	66 ± 7	62 ± 8	.014
BMI (kg/m^2^)	28 ± 4	26 ± 3	28 ± 4	.019
Female (%)	33 (28%)	11 (33%)	20 (25%)	.524
CHA_2_DS_2_‐VASc	2 [1–3]	2 [1–3]	2 [0‐3]	.345
COPD	13 (11%)	4 (12%)	8 (10%)	.543
Hemorrhage	5 (4%)	4 (12%)	0 (0%)	.002
OSAS	15 (13%)	2 (6%)	12 (15%)	.314
Vascular disease	10 (8%)	3 (9%)	7 (9%)	>.99
AF characteristics				
pAF	34 (29%)	12 (36%)	19 (24%)	.091
(Longstanding)‐persAF	85 (71%)	21 (64%)	62 (77%)	.091
AF duration (months)	61 [25‐125]	66 [23–128]	45 [26–119]	.533
AFL and/or AT	45 (38%)	7 (21%)	36 (44%)	.051
Previous CA ablation	53 (44%)	13 (39%)	39 (48%)	.669
Use of AAD I/III	70 (58%)	17 (52%)	44 (54%)	.115
Echocardiographic measurements				
LVEF (%)	54 ± 9	53 ± 10	54 ± 9	.860
LA (mm)	44 ± 7	45 ± 6	45 ± 7	.908
LAVI (ml/m^2^)	50 ± 16	51 ± 17	51 ± 15	.788
pAF	45 ± 14	45 ± 12	47 ± 17	.778
non‐pAF	52 ± 16	55 ± 19	52 ± 15	.438
RAVI (ml/m^2^)	38 ± 14	40 [22–49]	38 [28–46]	.121

Abbreviations: AAD, antiarrhythmic drug; AF, atrial fibrillation; AFL, atrial flutter; AT, atrial tachycardia; BMI, body mass index; CA, catheter ablation; COPD, chronic obstructive pulmonary disease; (D)OAC, (direct)oral anticoagulant; LA, left atrial; LAV, LA volume; LVEF, left ventricular ejection fraction; MI, mitral valve insufficiency; OSAS, obstructive sleep apnea syndrome; pAF, paroxysmal AF; RAV, right atrial volume; SVT, supraventricular tachycardia.

### 3.2 Procedural data

All 119 patients underwent unilateral left‐sided HA. In 10 patients (8%), epicardial ablation of the right PVs was incomplete due to a too‐short clamp (*n* = 6), no good visualization (*n* = 3), or bleeding of the RA (*n* = 1). Isolation of the PVs was attempted in all patients (*n* = 119) and the box in all but two (*n* = 117) that were known with paroxysmal AF (pAF). Furthermore, the LAA was occluded in 96% (*n* = 115) of all patients, either by AtriClip (90%, *n* = 103), Lariat (4%, *n* = 4), stapler (2%, *n* = 2), or a Watchman device (5%, *n* = 6). In the remaining five patients (4%), the LAA was not closed due to bleeding at its base (*n* = 1), CHA_2_DS_2_‐VASc Score of zero (*n* = 1), typical Wind‐sock configuration with a high risk of creating a rest pouch (*n* = 1), the inability of exposing the LAA due to adhesions after a previous coronary artery bypass surgery (*n* = 1) and AT ablation, not necessitating LAA closure (*n* = 1).

**Figure 1:**
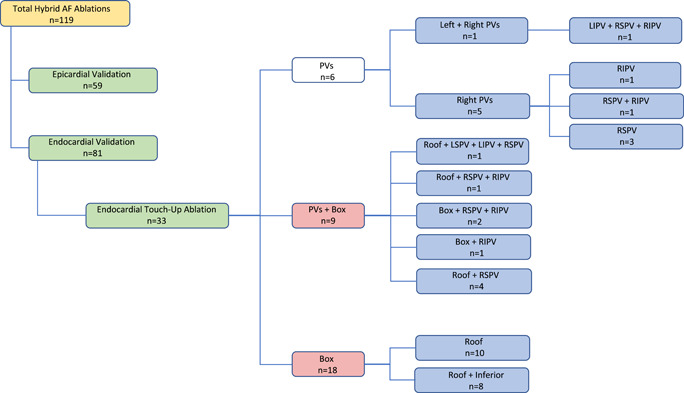
Overview of the number of patients undergoing endocardial touch‐up per region after epicardial ablation. AF, atrial fibrillation; LSPV, left superior PV; PV, pulmonary vein; RIPV, right inferior PV; RSPV, right superior PV.

**Figure 2:**
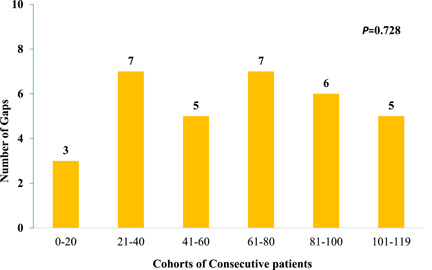
The number of unintended gaps following epicardial ablation for AF does not decrease over time. AF, atrial fibrillation.

For logistical and/or safety reasons, 81 of the 119 patients underwent an endocardial electrophysiology/electrophysiologist (EP) validation, 33 patients received epicardial EP validation only, and 5 patients received no EP validation. Of all patients undergoing endocardial validation, touch‐up of unintentional gaps was performed in 41% (*n* = 33/81) (Figure 1). Overall, 82% (27/33) of all touch‐up ablations concerned the box region, mainly in the roof line followed by the right PVs. To evaluate a possible influence of a “learning curve” on the number of endocardial touch‐up ablation needed, 6 cohorts of 20 consecutive patients were created (Figure 2). Over time, there was no significant difference in the number of unintended gaps between the cohorts (*p* = .728). In 48% of all patients (*n* = 58), additional endocardial ablation including a CTI line (*n* = 39), complex fractionated atrial electrograms (*n* = 15), and/or a mitral isthmus line (*n* = 8) with bidirectional block was performed. In 10 patients, a CTI line was created in a previous CA procedure and bidirectional block was still present during the HA procedure.

### 3.3 Complications

Perioperative major complications consisted of: bleeding requiring reoperation (*n* = 1, 0.8%), cardiac tamponade (*n* = 1, 0.8%), myocardial infarction requiring percutaneous coronary intervention and stenting of the left anterior descending artery (*n* = 1, 0.8%), pacemaker implantation due to conversion pauses (*n* = 1, 0.8%), and pneumothorax (*n* = 1, 0.8%). Minor complications were bleeding requiring transfusion or drainage (*n* = 4, 3.4%), hemodynamically stable pericarditis requiring medication (*n* = 1, 0.8%), and pneumonia (*n* = 1, 0.8%). There were no incidences of stroke, conversion to sternotomy, or mortality. Postoperative complications until 24 months of follow‐up were late cardiac tamponade (*n* = 2, 1.7%), diaphragm paresis (*n* = 1, 0.8%), hemothorax (*n* = 1, 0.8%), pacemaker or implantable cardioverter‐defibrillator‐implantation (*n* = 3, 2.5%), pericarditis requiring medication (*n* = 4, 3.4%), or pericardiocentesis (*n* = 2, 1.7%), hospital readmission due to decompensation cordis due to recurrent AF (*n* = 2, 1.7%), pleural effusion (*n* = 2, 1.7%), and pneumonia (*n* = 1, 0.8%) (Table 2).

**Table 2:** Peri‐operative complications and complications after discharge until 24 months following unilateral left‐sided thoracoscopic hybrid AF ablation

Peri‐operative	All patients (*n* = 119)
Bleeding requiring transfusion or drainage	4 (3%)
Bleeding reoperation	1 (1%)
Cardiac tamponade	1 (1%)
Myocardial infarction with PCI + DES LAD	1 (1%)
Pacemaker implantation	1 (1%)
Pericarditis (HD stable)	4 (3%)
Pneumonia	1 (1%)
Pneumothorax	1 (1%)
Total	14 (12%)
Until 24 months	
Cardiac rehospitalization	17 (14%)
Late cardiac tamponade ≤30 days	2 (2%)
Decompensation cordis due to AF	2 (2%)
Pericarditis OMT	4 (3%)
Pericarditis drainage	2 (2%)
Haematothorax	1 (1%)
Pacemaker or ICD implantation	3 (3%)
Pleural effusion	2 (2%)
Pneumonia	1 (1%)
Diaphragm paresis	1 (1%)
Total	18 (15%)

Abbreviations: AF, atrial fibrillation; DES, drug‐eluting stent; ICD, implantable cardioverter‐defibrillator; LAD, left descending artery; OMT, optimal medical treatment; PCI, percutaneous coronary intervention.

### 3.4 Follow‐up

**Figure 3:**
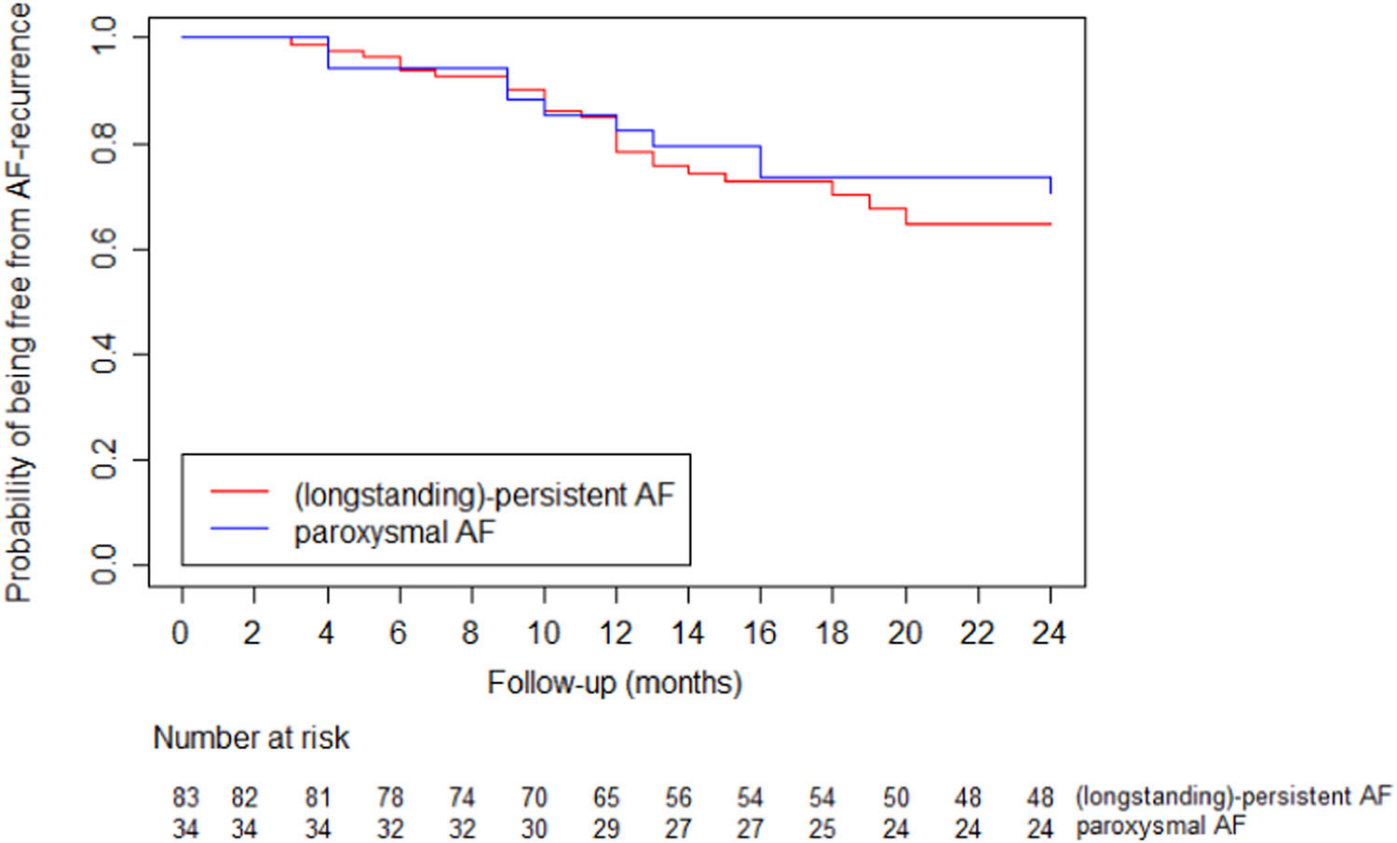
Kaplan–Meier curve depicting the probability of being free from AF recurrence following unilateral left‐sided hybrid AF ablation until 24 months of follow‐up based on preoperative rhythm (blue, pAF; red, non‐pAF). AF, atrial fibrillation; pAF, paroxysmal AF.

One and two‐year follow‐up was reached by 112 (94%) and 105 (88%) patients, respectively. Patients were lost to follow‐up as they had moved abroad or did not wish to receive further rhythm follow‐up. The primary outcome until 12 months was reached by 82% (allowing AADs) and 62% (off AADs) for pAF and 78% (allowing AADs) and 76% (off AADs) for (longstanding)‐persistent AF. After 2 years, the efficacy rates were 71% (allowing AADs) and 50% (off AADs) for pAF compared to 65% (allowing AADs) and 61% (off AADs) for (longstanding)‐persistent AF (Figure 3, Table 3). When comparing the efficacy outcome between patients undergoing epicardial validation only and endocardial validation, no significant difference was found (all patients *p* = .584, pAF *p* = .920, persistent AF *p* = .385) (Supporting Information: Table S1). Furthermore, the discontinuation of AADs of all patients who were in sinus rhythm (SR) after 12 months did not differ between pAF (83%) and non‐pAF (78%) (*p* = .574) nor after 24 months (pAF 86%, non‐pAF 88% *p* = 1.000) (Supporting Information: Figure S1).

**Table 3:** Efficacy rates of a unilateral left‐sided hybrid ablation

Efficacy outcomes		SR allowing AADs	SR off AADs
12 months	All patients	80% (73–87)	72% (64–81)
	pAF	82% (71–96)	62% (47–81)
	non‐pAF	78% (70–88)	76% (67–86)
24 months	All patients	67% (58–76)	58% (49–68)
	pAF	71% (57–88)	50% (36–70)
	non‐pAF	65% (55–77)	61% (51–73)

*Note*: Groups were made based on preoperative rhythm.

Abbreviations: AAD, antiarrhythmic drug; AF, atrial fibrillation; pAF, paroxysmal AF; SR, sinus rhythm.

## 4 DISCUSSION

In this study, we describe the efficacy and safety of a unilateral left‐sided thoracoscopic hybrid AF ablation approach in a cohort study of 119 patients. After 12 and 24 months, freedom from supraventricular arrhythmia recurrence was 80% and 67%, respectively, when allowing AADs. Major and minor complications during the hospital stay and after discharge until 24 months were low.

### 4.1 Efficacy

While unilateral thoracoscopic beating‐heart approaches with microwave or unipolar radiofrequency (RF) have previously been reported,^7^ thoracoscopic AF ablation using a bipolar RF clamp is usually performed via a bilateral approach.^2^, ^8^ More recently, two unilateral techniques have been introduced: a left‐sided technique, either as a stand‐alone procedure^5^, ^9^ or concomitant to minimally invasive bypass grafting of the left anterior descending artery,^10^ and a right‐sided epicardial approach.^11^ The quest for unilateral thoracoscopic approaches is driven by the conceivably advantages such as reduced surgical trauma and less complications on the contralateral side.

In our study, freedom from atrial tachyarrhythmia recurrence after 12 months was 88% in pAF and 73% in non‐pAF, when allowing AADs. Compared to de Asmundis et al.,^9^ who reported on 51 patients undergoing unilateral left‐sided HA ablation with 69% of patients in SR off AADs after 24 months, our study showed a comparable overall efficacy rate of 65%, but on AADs. Moreover, Pison et al.^2^ reported on 1‐year efficacy rates following a bilateral approach of 93% in SR for pAF versus 90% in SR for non‐pAF, when allowing AADs. Maesen et al.^3^ even reported on efficacy results 3 years after a bilateral HA approach, with success ratios of 80% and 79% of patients in SR for pAF and non‐pAF, respectively, both with and without the use of AADs. Both our 1 and 2 years results seem lower than the results published by Pison and Maesen et al. There are several explanations for this difference. First, our patient population was more difficult to treat, as they had higher CHA_2_DS_2_‐VASc scores, larger LA and more patients had (longstanding)‐persistent AF. Second, discontinuation of AADs was left to the discretion of the referring cardiologists and AADs were often continued for unknown reasons.

Within the hybrid concept, several surgical techniques have been described to improve outcomes.^12^ In the CONVERGE trial,^13^ non‐pAF patients were randomized between the hybrid convergent technique (posterior LA wall isolation with a vacuum‐assisted unipolar RF catheter and endocardial PV isolation (PVI) to complete the box lesion set) or a CA approach. Although the efficacy outcome in the hybrid convergent arm was significantly higher compared to the catheter arm (67.7% vs. 50.0% when allowing the use of AAD), the arrhythmia‐free survival at 1 year was low compared to our study. The advantage of the convergent technique is that is allows access via a minimally invasive subxyphoidal approach, while its disadvantage is that is offers poor efficacy results (53% off AAD after 1 year). This may be explained by the fact that unipolar RF is used, pulmonary vein isolation had to be completed with CA and due to the absence of LAA occlusion.

Another mono‐lateral technique as part of a two‐stage hybrid approach uses a flexible RF device (COBRA Fusion, AtriCure Inc.) to encircle the 4 PVs and perform posterior LA isolation via right‐sided thoracoscopy, followed by an endocardial validation after 3 months.^7^ Efficacy outcomes following the epicardial ablation were poor, as only 50.6% presented in sinus rhythm before the second stage CA. After the endocardial touch‐up, efficacy rates improved to 63% off AAD, which again highlights the importance of a hybrid approach.^14^ Although the technique uses a combination of unipolar and bipolar RF, its success is strongly dependent on suction‐dependent tissue contact and, therefore, less effective that the bipolar biparietal RF application.^15^ Recently, the technique of a totally thoracoscopic right‐sided AF approach using a bipolar RF clamp was described in 13 patients.^11^ As the authors did not report on efficacy and safety outcomes after 3 months follow‐up, we cannot compare both unilateral approaches after 1 and 2 years. Nevertheless, a left‐sided approach has several potential advantages over a right‐sided approach: larger lung capacity during single right lung ventilation, dissection of the pericardial folds away from the heart, adequate visualization of the LAA during exclusion and PVI with the convex side of the bipolar clamp toward the heart, resulting in antral isolation and minimal risk of PV stenosis. The right‐sided approach, on the other hand, has the advantage of adding RA lines. However, it remains to be determined if these RA lesions are beneficial without its key lesion (the lesion toward the tricuspid annulus). From an electrophysiological point of view, only a circular lesion to isolate the superior caval vein has been proven to increase the success rate, especially in patients with (longstanding)‐paroxysmal AF who present with trigger‐initiated AF rather than substrate perpetuation of AF.^16^

While the use of a biparietal bipolar RF clamp for PVI is thought to be very effective in creating transmural lesions,^17^ even bipolar RF energy cannot always prevent conduction gaps.^18^ As such, the natural strength of a HA is that epicardial ablation is succeeded by endocardial validation of bidirectional conduction block and the possibility of precise touch‐up of unintentional gaps.^2^ Especially for a unilateral (left‐sided) thoracoscopic procedure, establishing bidirectional conduction block is of paramount importance, as it can be challenging to reach and completely isolate the right PVs. While the epicardial conduction block of the PVs and box corresponds well with the endocardial conduction block, the completeness of right superior PV (RSPV) isolations and linear lesions is often misjudged.^19^ This may be due to tissue edema, an antral location of the conduction gap, or misplacement of the mapping catheter due to limited visualization in a unilateral approach.^19^ Therefore, the added value of the endocardial ablation and ‐validation to prevent false negative results following epicardial ablation and ‐validation should not be underestimated. Furthermore, RSPV ablation specifically can be challenging because of anatomy or adhesions and could explain that the majority of endocardial touch‐up ablation were in the region of RSPV and the adjacent superior part of the box lesion. This can be partly overcome by verifying the position of the RF clamp on the RSPV via the transverse sinus during the isolation of the right veins. Conduction gaps at the junction of the roof line and RSPV, potentially associated with this unilateral approach, can easily be addressed from the endocardium within the hybrid setting. As such, the need for a right thoracoscopy can be avoided, thereby reinforcing the need for endocardial EP validation following a unilateral left‐sided HA. In our study, patients were preselected based on logistical and/or safety reasons to either undergo epicardial and/or endocardial EP validation. For example, patients with a previous hemorrhage underwent epicardial validation to prevent the need for full heparinization, whereas patients with a high chance of failure due to a higher substrate, for example, obesity and higher BMI, underwent endocardial validation. In a subset of patients, no EP validation was performed at all due to safety reasons such as procedural bleeding.

While PVI is believed to be the cornerstone in AF ablation,^20^ PV isolation alone is insufficient to achieve satisfactory results in more persistent forms of AF. Other non‐PV sources of arrhythmias of the LA include the appendage. Not only did the LAAOS‐III trial indicate that concomitant LAA occlusion significantly reduces the risk of stroke in patients with AF undergoing cardiac surgery,^21^ the BELIEF trial showed that electrical isolation of the LAA also improves long‐term freedom from supraventricular tachyarrhythmias.^22^ Other LA anatomical structures that are often involved in re‐entry leading to atrial tachyarrhythmias, which may, therefore, represent important targets for AF substrate ablation, include Bachman's bundle,^23^ the Ligament of Marshall, and the coronary sinus.^24^

### 4.2 Complications

Inherently, although minimally invasive, surgical techniques come with higher complication rates than truly minimally invasive CA techniques. In our study, major and minor complications during hospitalization occurred in 12% of patients, although the severity of pneumothorax after thoracoscopic surgery remains semantic. Not unimportantly, pericarditis without hemodynamic instability and bleeding requiring transfusion or drainage represented half of all complications. Major complications occurred in 4% of all patients and there were no incidences of conversion to sternotomy, atrio‐esophageal fistula, stroke, or mortality. Compared to the incidence of major complications after percutaneous approaches, which are also around 5%,^25^ our complication rate was similar. After discharge, complications occurred in 18 patients (15%) until a long‐term follow‐up of 24 months. In a safety analysis of thoracoscopic surgical ablation only, the 30‐day complication rate including peri‐operative complications was already 12%.^26^ As such, although perhaps technically more challenging, the shift to a left‐sided only approach may even reduce the rate of major and minor complications until long‐term follow‐up. Importantly, not only acute complications must be taken into account, but also the added risk of complications for every additional ablation in repeat procedures to establish freedom from AF recurrence.^27^ Therefore, unilateral left‐sided thoracoscopic HA may not only reduce major complications compared to a bilateral approach but also to percutaneous approaches on the long term. Furthermore, as surgical ablation for AF improves the quality of life (QOL), especially in patients where SR is established,^28^ QOL may even further improve when complications and postoperative pain are reduced by limiting surgery to one side only.

## 5 LIMITATIONS

Our data are derived from a single‐center observational study, where all procedures were performed by an experienced team, thereby reducing the generalizability of the study. Second, not all patients underwent endocardial validation and some patients received no EP validation at all. According to the definition of a HA, it is questionable whether these patients received a “true” HA procedure. Further limitations are the lack of structural rhythm follow‐up until 24 months and a protocol mandating discontinuation of AADs, which affects the efficacy results off AAD.

## 6 CONCLUSION

Within the concept of a single staged HA for AF, a unilateral left‐sided thoracoscopic approach for patients with pAF or persAF is efficacious and safe. A randomized controlled trial comparing the unilateral left‐sided hybrid approach with bilateral approaches or with stand‐alone percutaneous and/or epicardial approaches should be performed to validate our results.

## CONFLICT OF INTEREST

B. M., J. L., and M. L. M. are consultants for AtriCure and/or Medtronic.

## Supporting information

Supplementary information.
