# Supplementary material for: Hybrid ablation of atrial fibrillation: A unilateral left‐sided thoracoscopic approach
Source: J Card Surg. 2022 Nov 9;37(12):4630–8. doi: 10.1111/jocs.17144 (PMC10099869; doi:10.1111/jocs.17144)
Supplement: Supplementary file 1 — Supplementary information. [file JOCS-37-4630-s001.docx]

**Supplements**

**Figure S1.** Comparison of patients that were free from AF recurrence when allowing AADs and off AADs, after 12 and 24 months. **(A)** Patients with pre-operative paroxysmal AF. **(B)** Patients with pre-operative (longstanding)-persistent AF. AAD=antiarrhythmic drugs; AF=atrial fibrillation.

**Table S1.** Efficacy rates of a unilateral left-sided hybrid ablation. Groups were made based on electrophysiological validation strategy and pre-operative AF type. pAF=paroxysmal AF.

**Table S1.** Efficacy results until 12 and 24 months after unilateral left-sided hybrid ablation based on electrophysiologic validation strategy.

| SR allowing AADs | | Epicardial validation | Endocardial validation |
| --- | --- | --- | --- |
| 12 months | All patients | 87% (77 – 100) | 75% (66 – 86) |
|  | pAF | 84% (70 – 100) | 74% (64 – 86) |
|  | non-pAF | 92% (77 – 100) | 79% (63 – 77) |
| 24 months | All patients | 70% (56 – 89) | 65% (55 – 76) |
|  | pAF | 62% (43 – 89) | 63% (52 – 77) |
|  | non-pAF | 83% (65 – 100) | 68% (50 – 93) |
